# Viewing sexual images is associated with reduced physiological arousal response to gambling loss

**DOI:** 10.1371/journal.pone.0195748

**Published:** 2018-04-12

**Authors:** Ming Lui, Ming Hsu

**Affiliations:** 1 Department of Education Studies, Hong Kong Baptist University, Hong Kong, China; 2 Haas School of Business, University of California, Berkeley, California, United States of America; Technion Israel Institute of Technology, ISRAEL

## Abstract

Erotic imagery is one highly salient emotional signal that exists everywhere in daily life. The impact of sexual stimuli on human decision-making, however, has rarely been investigated. This study examines the impact of sexual stimuli on financial decision-making under risk. In each trial, either a sexual or neutral image was presented in a picture categorization task before a gambling task. Thirty-four men made gambling decisions while their physiological arousal, measured by skin conductance responses (SCRs), was recorded. Behaviorally, the proportion of gambling decisions did not differ between the sexual and neutral image trials. Physiologically, participants had smaller arousal differences, measured in micro-siemen per dollar, between losses and gains in the sexual rather than in the neutral image trials. Moreover, participants’ SCRs to losses relative to gains predicted the proportion of gambling decisions in the neutral image trials but not in the sexual image trials. The results were consistent with the hypothesis that the presence of emotionally salient sexual images reduces attentional and arousal-related responses to gambling losses. Our results are consistent with the theory of loss attention involving increased cognitive investment in losses compared to gains. The findings also have potential practical implications for our understanding of the specific roles of sexual images in human financial decision making in everyday life, such as gambling behaviors in the casino.

## Introduction

In the past decades, the role of emotions in choice behaviors has been a hot topic in the decision-making research community. Erotic images are emotional signals frequently encountered in daily life, such as in advertisements, websites, TV shows, and movies. Like hunger and thirst, sexual desire creates one of the strongest motivations for human behaviors, along with other appetitive functions in the brain [1]. Nevertheless, few studies have examined how sexual stimulation influences human decision-making behaviorally and physiologically. In a study by Ariely and Loewenstein (2006) [2], men in a sexually aroused state had a higher tendency to accept unsafe sex and morally questionable behaviors. Another field study [3] showed that young male skateboarders took higher physical risk of crash landings in the presence of an attractive female compared to a less attractive female, an effect associated with an increased level of testosterone. In addition to risky sexual and physical activities, evidence has accumulated regarding the effect of sexual stimuli on financial decision-making such as delayed discounting [4, 5], the ultimatum game [6], the Iowa Gambling Task [7], and financial risk-taking [8].

These studies revealed that sexual stimuli were associated with heightened desire for immediate reward [4] and a higher tendency toward risk-taking [8]. Findings on the effect of sexual arousal on financial decision-making have remained scarce, and none of the past studies has examined loss aversion. *Loss aversion* was first described by Kahneman and Tversky [9] as the overweighting of losses compared to equally large gains [10], and is thought to be a hallmark of irrational decision-making that involves emotional processes. In addition to behavioral findings (e.g. [11]), there were physiological (such as pupil diameter, heart rate, and skin conductance) and brain imaging data demonstrating that losses had a larger impact than gains on our emotional and cognitive systems (e.g. [10, 12]). The concept of loss aversion is distinct from *risk-seeking*, which is characterized by increased marginal utility of money. Some past findings suggest that loss aversion has a stronger effect on modulating decision-making behavior than risk-seeking (see [13] for a review). However, there is also evidence showing no loss aversion among average decision makers [14, 15, 16], even with the presence of heightened physiological responses (such as pupil dilation and increased heart rate) to losses compared with gains [17, 18]. Given the inconsistent findings, Yechiam and Hochman [18] have recently proposed an attentional model of loss regarding the impact of loss on behavioral decisions and physiological arousal (e.g. [17]). The model proposes that losses lead to increased physiological arousal as well as vigilance to task, causing an increase in sensitivity to the outcomes of decisions including both losses and gains. This in turns promotes behavioral responses according to the reinforcement structure and reduces random behaviors.

Regarding the effect of sexual stimuli on decision making, some past studies have shown that sexual stimuli interferes with attention and leads to distraction from the task requirements [19, 20], thereby reducing the mental resources for processing emotional responses associated with gain and loss outcomes [21, 22]. On the other hand, neuroscientific findings have shown that sexual stimuli activate the dopaminergic circuitry of the brain reward system, such as the nucleus accumbens, similarly to other types of rewards such as money and drugs [23]. Dopamine in the nucleus accumbens is responsible for motivational and approach behavior for reward-seeking (see [24] for a review). The activation of the appetitive neural system by sexual stimuli may account for the heightened desire for immediate monetary reward [4] and a higher tendency of financial risk taking [8] when people are sexually aroused, as found in previous studies. Given that past findings of the effects of sexual arousal on decision-making behaviors remain scarce, our study aims to fill this research gap by examining the effect of sexual images on decision-making under risk. The prospect theory proposes that people make real-life decisions based on the potential values of losses and gains when the probabilities of outcomes are known [9]. In our experiment, participants were informed that their decisions to gamble would result in 50% chance of monetary gain and 50% chance of monetary loss of known values. Immediately before this gambling task, participants were required to evaluate pictures with either erotic or neutral content, which was designed to manipulate participants’ level of sexual arousal. They were told that the two tasks were separate experiments. Our study adopted a within-subject repeated-measures design because of the large between-subject variations in risk attitude, which can substantially reduce the power of studies. In fact, within-subject designs have long been used by decision-making researchers to mitigate this variation and have also been increasingly recommended in other areas of psychology (Normand, 2016).

Regarding the dependent variables, participants’ behavioral responses (decisions of gambling and reaction time) and physiological responses (skin conductance responses; SCRs) were measured. We examined both the decisions and the reaction times because we believed that sexual stimulation may affect participants’ decisions to gamble and the time required for the decisions to be made. Reaction time reflects the level of mental conflict between gambling and not gambling during the decision making process [25]. SCR is an index of autonomic arousal triggered by the sympathetic nervous system and we measured participants’ arousal responses to sexual image viewing and to the outcome of gambling (i.e. monetary gains and losses) through the measurements of SCRs time-locked to the onsets of these stimuli.

In terms of behavioral results, we hypothesized that sexual stimuli would activate the reward system and stimulate the appetitive motivation to seek potential rewards, which would manifest as a higher proportion of gambling decisions in sexual image trials for all risk ratios. Increased appetitive motivation was consistently found to cause people to have diminished sensitivity to risks; for example, drug addicts during craving (Naqvi et al., 2014) and risky sexual behaviors (Ariely & Loewenstein, 2006). Therefore, we proposed that people would demonstrate a higher proportion of gambling in all risk-ratios. Second, the increased appetitive motivation in sexual trials may lead to a reduction in the mental conflicts between the decisions (gambling or not gambling) [25], which may in turn cause a faster reaction time in sexual image trials relative to neutral image trials. Third, we hypothesized that sexual stimulation would attenuate participants’ emotions triggered by gambling losses relative to gains: they would have lower SCRs to gambling losses relative to gains in sexual than in neutral image trials. This hypothesis was based on the neuroscientific findings that sexual stimuli are associated with the release of endogenous opioids [26], leading to a euphoric state and thus might buffer against the negative emotional arousal triggered by monetary loss from gambling. In other words, we hypothesized that sexual stimulation would reduce loss aversion, both behaviorally and physiologically. Finally, we hypothesized that participants’ SCRs to losses relative to gains would predict gambling decisions, that is, participants who had higher physiological arousal to losses relative to gains would gamble less, based on Sokol-Hessner et al. (2009)’s finding of correlation between behavioral loss aversion and SCRs to losses relative to gains [12].

## Materials and methods

### Participants

Thirty-eight heterosexual males participated in the experiment. Four participants’ data were excluded because of lack of SCR responses during the deep-breath test (N = 3), or a technical error (N = 1). The final data set contains 34 subjects, aged between 18 and 27 years (*M* = 21.5; *S*.*D*. = 2.3), reporting no neurological or psychiatric conditions. All participants were considered right-handed based on the Edinburgh Handedness Inventory [27]. Before the participants joined the experiment, they completed an online version of the Autism Spectrum Quotient (AQ) [28], which is a self-administered instrument for measuring the degree of autism traits among adults with normal intelligence. Since there was evidence showing diminished neural and behavioral responses to monetary reward among individuals with autism traits (e.g. [29]), only participants with AQ lower than 30 were included in this study. Data of participants with high AQ were analyzed separately to be included in another study.

Our study has received approval from the Committee on the Use of Human and Animal Subjects in Teaching and Research (HASC) at Hong Kong Baptist University (approval number: FRG1/14-15/067). All procedures performed in this study were in accordance with the ethical standards of HASC and with the 1964 Helsinki declaration and its later amendments or comparable ethical standards. Written informed consent was obtained from all participants. The payment scheme was explained to the participants thoroughly before the experiment, and all participants were reimbursed accordingly.

### Psychophysiological measures

Participants’ skin conductance responses (SCRs) were recorded by the eegosport system (ANT Neuro Ltd.) connected to a laptop computer. Triggers were sent from the stimulus presentation program (Eprime) of one computer to the SCR signal recording software in another computer. Electrodes made of Ag-AgCl were attached to the palmar surface of participants’ index and middle fingertips of their left hand. The SCR data were event-related measures, which were time-locked to the triggers sent at the onset of the images and of the gambling outcomes. The SCR data were sampled at 500 Hz, low-pass filtered (20 Hz), smoothed (9 sample kernel), transformed from kOhm to micro-siemens (μ S), and square-rooted using an in-house MATLAB script. The SCRs were the baseline-to-peak amplitude difference in the 0.5-s to 5-s time window from the stimulus-onset. The physiological data of 4 participants who had extremely low gambling responses (< = 15%) were excluded because of too few trials per condition for analysis. These 4 participants’ behavioral data, including decisions and reaction times, were included in the behavioral data analysis only.

### Procedure

At the beginning of the experiment, participants were told that they would perform two separate experiments; one was a picture categorization task, and the other was a financial decision task. In the picture categorization task, participants saw pictures of young women. The female pictures were selected based on ratings previously given by 20 male heterosexual college students (mean age = 22.4) who did not participate in this current decision-making experiment. The mean arousal ratings (with a 9-point Likert scale) of sexy and neutral pictures were 6.21 (S.D. = .25) and 1.71 (S.D. = .26) respectively. The proportions of sexual and neutral female pictures were equal (.5 vs .5) and the order of presentation was randomized. With a response box, participants pressed the “+” button if they regarded the picture as sexy; they pressed the “0” button if they regarded the picture as neutral. Positions of the buttons were counterbalanced across participants. They were instructed to press the button with a response box within a time limit of 1.5 seconds. Responses made beyond the time limit were not recorded.

**Fig 1:**
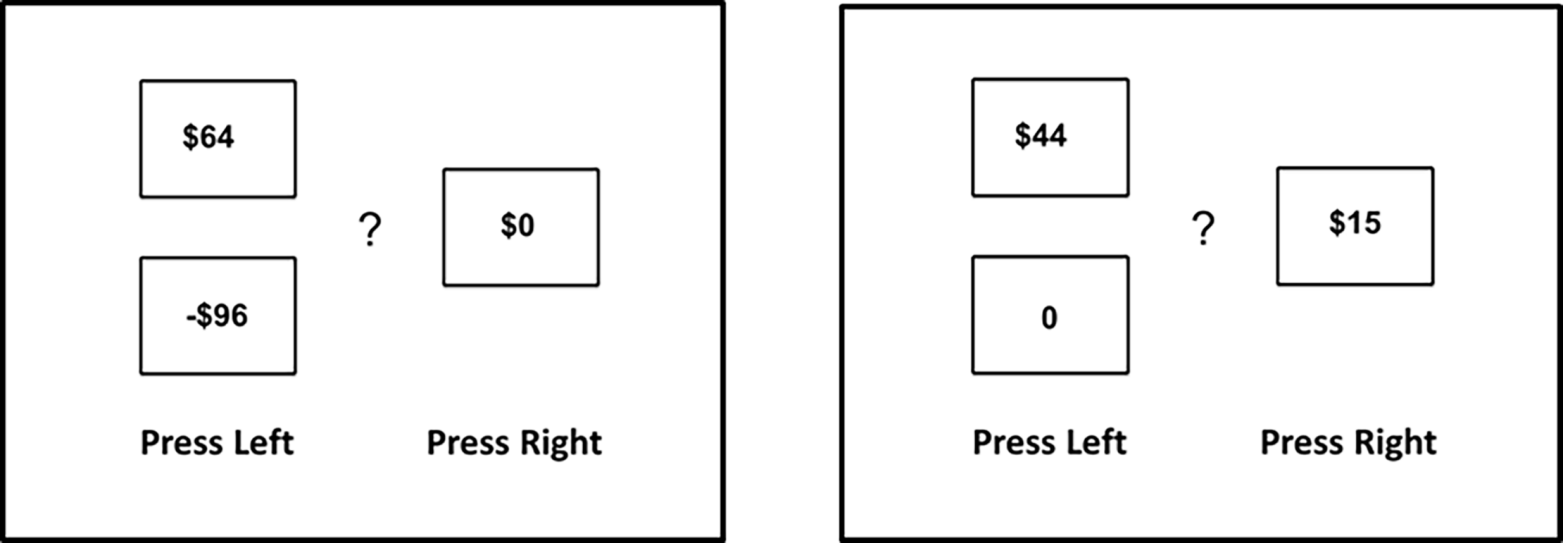
**(Left panel) The two options in gain-loss trials.** The participants chose either the left or right option. In this example, there is a 50% chance of gaining HKD $64 and a 50% chance of losing HKD $96 if the left option is chosen. If the right option is chosen, there will not be any loss or gain. **(Right panel) The two options in the gain-only trials.** In this example, there is a 50% chance of gaining HKD $44 and a 50% chance of gaining nothing ($0) if the left option is chosen. If the right option is chosen, the outcome is a certain gain of HKD $15.

For the financial decision task, before the start of the experiment, participants were given HKD $50 cash for gambling and were told to put it in their wallet. During the task, the participants were required to choose between two options that involved different levels of financial risk. Referring to an example of gain-loss trial (Fig 1 left panel), participants were told that if they chose the *left* option, there was a 50% chance of gaining the monetary amount shown at the upper left corner and a 50% chance of losing the amount shown at the lower left corner. If the participant chose the *right* option, it was 100% certain that they would receive the amount indicated on the right, which could be zero or a positive number (please refer to S1 File of supporting information for the complete set of instructions). Table 1 show the possible gain and loss combinations of all 72 trials. Seventy-two trials were gain-loss trials (Table 1) in which there was a 50% chance of losing or gaining money if the participant chose the left option (Fig 1 left panel). To compare the same choice options under different conditions of sexual arousal, each gain-loss combination was presented twice, once after a sexual image and once after a neutral image. The 36 combinations therefore resulted in 72 gain-loss trials (Table 1). There were eight gain-only choices (i.e. 8 choices x 2 conditions = 16 trials) in which the left option led to either a positive or zero outcome (50% chance), and the right option led to a 100% chance of gain (Fig 1 right panel). The gain-loss trials and gain-only trials were randomly presented for each participant. The gain-only trials were for identifying the model parameters to capture risk aversion. They were included for a close replication of the choices in Sokol-Hessner et al. (2008), which measured loss aversion and cognitive regulation. We did not include any analysis on these gain-only trials because the present study focused on model-free comparison and the gain-only trials are not directly comparable with the gain-loss trials.

**Table 1:** Gain-loss trials: Combinations of possible gains and losses leading to different risk ratios. Each gain-loss combination was presented twice, once after a sexual image and once after a neutral image. The 36 combinations contributed to 72 gain-loss trials.

	Possible Gain					
Risk ratio	44	48	52	56	60	64
0.25	-11	-12	-13	-14	-15	-16
0.5	-22	-24	-26	-28	-30	-32
0.75	-33	-36	-39	-42	-45	-48
1	-44	-48	-52	-56	-60	-64
1.25	-55	-60	-65	-70	-75	-80
1.5	-66	-72	-78	-84	-90	-96

**Fig 2:**
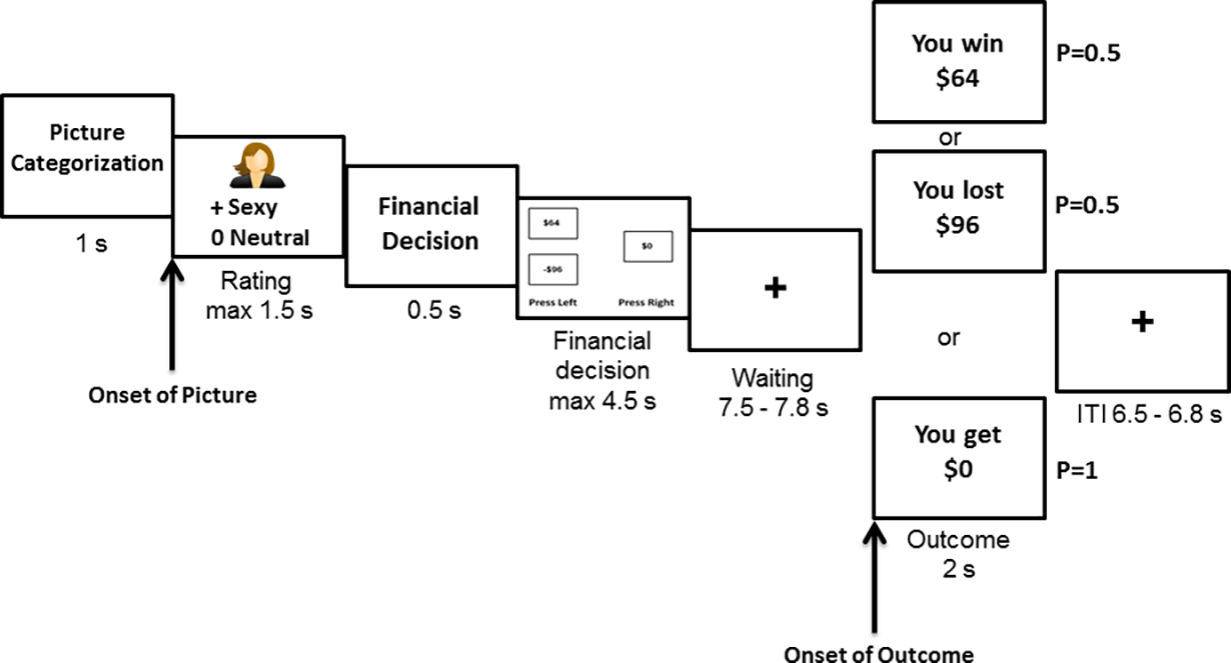
Stimulus events in a single trial.

Please refer to Fig 2 for the timings of events in each trial. Participants were required to respond within a 4.5-second time window; however, they were not told to respond as fast as possible. Responses made beyond the time limit were not recorded. If the participant did not respond within the time limit, the outcome screen would show the feedback “You were too slow”. The outcome of each participant’s decision was shown after a period of waiting (7.5–7.8 s). Note that a time gap was inserted between the financial decision screen and the outcome screen, as well as between trials (6.5–6.8 s). This was so that the skin conductance level could return to the baseline before the onset of the next stimulus.

Before the experiment, the participants were clearly informed that after they completed all 88 trials, eight of the trials would be randomly selected for calculation of the final amount of payment they would receive. The net gain or loss from the eight selected trials would be added to or subtracted from the HK$50 cash they received at the beginning. If the net amount was less than zero, they only needed to return all the HK$50 and no extra money would be requested. According to this calculation method, the maximum possible monetary reward a participant could receive was HK$586 and the minimum was HK$0. At the end of the experiment, the participants were asked the following four questions in an interview: 1) Do you have any ideas about these two experiments? 2) What do you think are the objectives of these experiments? 3) What strategy have you applied in the financial decision-making task? 4) Have you ever participated in a similar experiment? If so, please describe the procedures.

## Results

### Behavioral findings

As a manipulation check to see whether participants found the sexual images sexy and the neutral images neutral, participants’ responses to the picture categorization task were analyzed. Thirty-one out of the 34 participants rated over 80% of the sexual images as sexy and over 80% neutral images as neutral. We analyzed the behavioral and physiological data excluding the three participants who did not have over 80% responses consistent with the intended stimulus manipulations. The patterns of results were identical to the analyses with all participants’ data included. We therefore included all participants’ data in the statistical analyses without exclusion based on results of the picture categorization task.

The outcomes of gains and losses were fully randomized across sexual and neutral image trials by the stimulus presentation software. The 34 participants gambled in 1185 trials out of the 2445 gain-loss trials. The numbers of gain and loss outcomes in sexual and neutral image trials were 285 (gains in sexual trials), 317 (losses in sexual trials), 296 (gains in neutral trials) and 287 (losses in neutral trials) respectively. The chi-square statistic was 1.39 (*p* = .24), showing no significant difference in the proportions of gains and losses in the sexual and neutral image trials.

**Fig 3:**
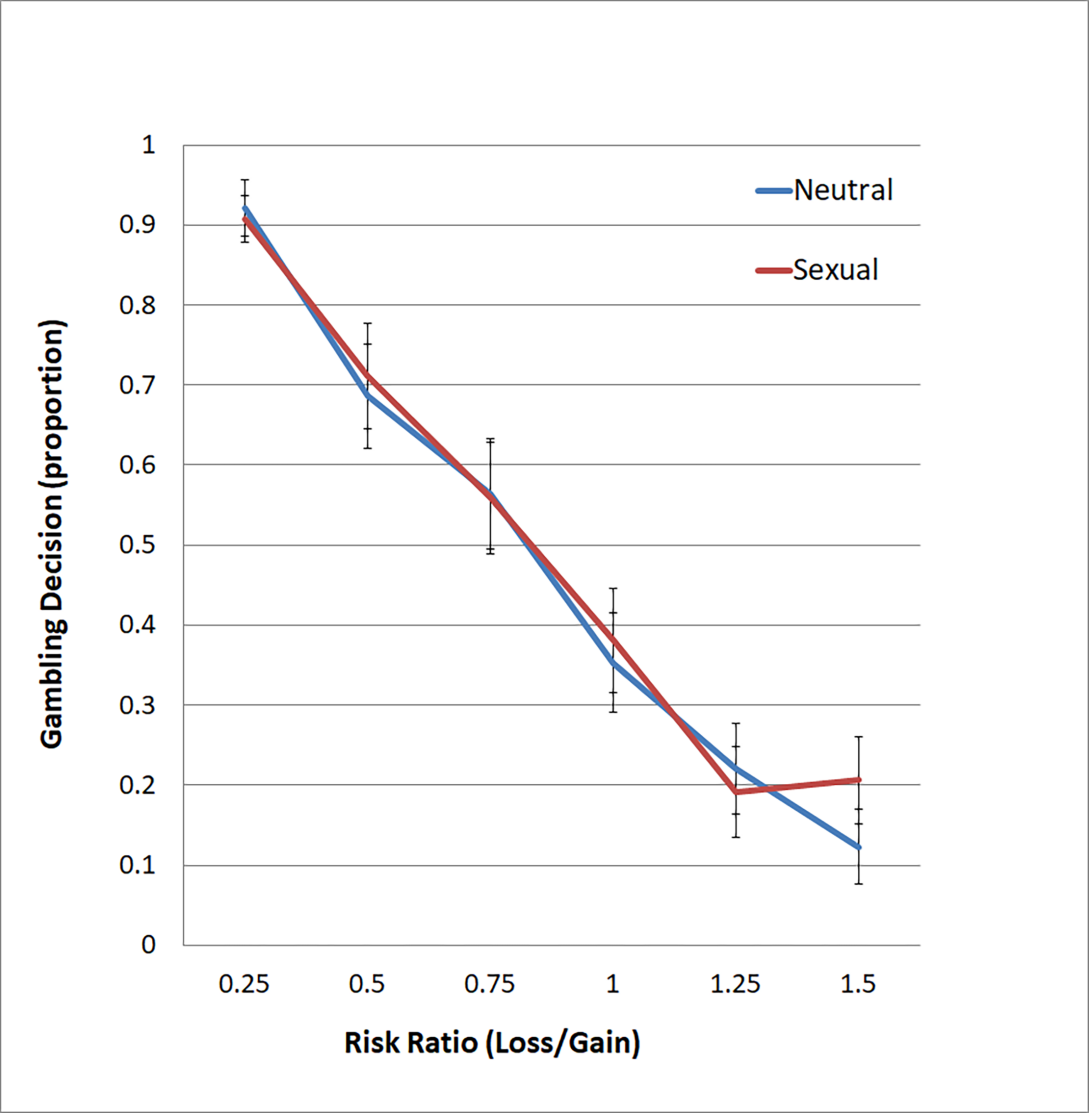
Proportions of gambling decisions made in trials with different risk ratios (.25–1.5) under sexual and neutral conditions.

The proportions of gambling decisions for choices with risk ratios of .25, .5, .75, 1, 1.25 and 1.5 were .91, .70, .56, .37, .21, and .16 respectively (averaging across the sexual and neutral trials). The low proportions (.56, .37, .21, and .16) of gambles in trials with risk ratios of .75 and higher indicated loss aversion among participants. Two-way repeated-measures ANOVAs were conducted to examine the effects of image (sexual vs. neutral) and risk ratios (five ratios from .25 to 1.50) on gambling decision and reaction time. Greenhouse-Geisser corrections were applied to effects with significant results of Mauchly’s Test of Sphericity. For gambling decisions, there was a significant main effect of risk ratio (*F*(2.81, 92.68) = 61.93, *p* < .001, partial *η*^2^ = .65). Pairwise comparisons showed that participants had significantly fewer gambles in the trials with higher risk ratios compared to trials with lower risk ratios (*ps* < .001), except for the comparison between 1.25 and 1.5 risk ratio (Fig 3). The effect of image (sexual vs. neutral) was not significant (*p* = .25), nor was the interaction between image and risk ratios (*p* = .15). Referring to Fig 3, it is apparent that participants made similar proportions of decisions to gamble in trials with all risk ratios except for the highest risk ratio, 1.5. Paired sample t-tests (with Bonferroni corrections) showed that the number of gambles did not differ between sexual image trials and neutral image trials for all risk ratios; the difference was close to the point of significance for trials at the highest risk ratio of 1.5 (*t(*33) = 2.70, *uncorrected p-value =* .*011*, *corrected p-value =* .*066*, partial *η*^2^ = .18).

There was a significant main effect of risk ratio for reaction time (*F*(3.10, 102.32) = 3.75, *p* = .013, partial *η*^2^ = .10). Descriptive statistics showed that participants responded the fastest (*M* = 1.05 s; *SD* = .043) in trials with the lowest risk ratio of .25, and they responded the slowest in trials with a risk ratio of .75 (*M* = 1.24 s; *SD* = .070). No other main effect or interaction was significant.

### Physiological findings

**Fig 4:**
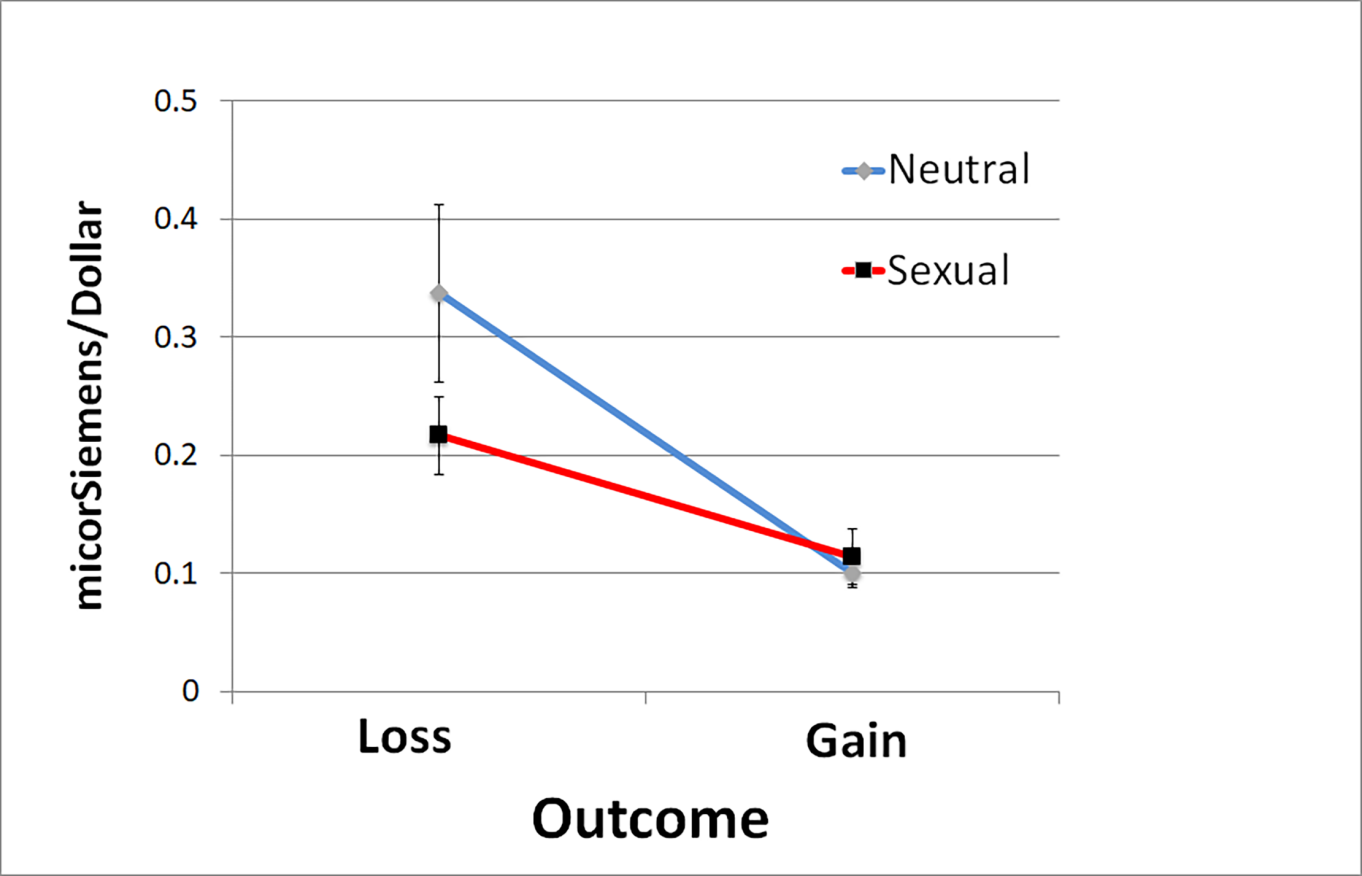
Skin conductance responses (in micro-siemen per dollar) to outcome of gambling losses or gains under sexual or neutral image trials.

Physiological arousal responses (in terms of μS/$) to the outcome of gambling were measured by the baseline-to-peak amplitude difference in the 0.5-s to 5-s time window from the onset of the gambling outcome screen. To examine the effect of sexual stimuli on physiological arousal to gambling outcome, a two-way repeated-measures ANOVA was conducted with image (sexual vs. neutral) and outcome (losses vs. gains) as the factors (Fig 4). There was a significant main effect of outcome: the participants had significantly larger arousal to gambling losses than to gambling gains (*F*(1, 29) = 16.92, *p* < .001, partial *η*^2^ = .37). There was a trend that participants had smaller arousal to gambling outcome in sexual than in neutral image trials (*F*(1, 29) = 3.69, *p* = .065, partial *η*^2^ = .11). The interaction between image and outcome was significant (*F*(1, 29) = 4.21, *p* = .049, partial *η*^2^ = .13). A post-hoc paired samples t-test showed that participants had significantly smaller arousal difference between losses and gains in sexual than in neutral image trials (*t(*29) = 2.05, *p* = .049, partial *η*^2^ = .13). To examine whether the effect found was due to differences in the initial skin conductance level at the onset of gambling outcome (the baseline), a paired sample t-test was conducted. No significant difference was found between sexual and neutral image trials at baseline (*t(*29) = .11, *p* = .915). The result suggested that the smaller arousal difference between losses and gains in the sexual than in the neutral trials was not due to any initial difference in skin conductance level at the baseline (i.e. the onset of release of gambling outcome).

Furthermore, linear regression analyses were conducted to examine whether the SCRs (in terms of μS/$) to losses relative to gains predicted participants’ proportion of gambling decisions made in neutral and sexual image trials. The SCRs to losses relative to gains were calculated by SCRs to losses divided by SCRs to gains. The SCRs to losses relative to gains significantly predicted the proportion of gambling decisions in the neutral image trials (standardized coefficients *β* = -.50, *t* = -3.07, *p* = .005; Adjusted *R*^2^ = .23) but not in the sexual image trials (standardized coefficients *β* = -.13, *t* = -.70, *p* = .49; Adjusted *R*^2^ = -.018).

Finally, to investigate habituation, we split the trials into first half and second half to check whether there was any change in participants’ behavioral decisions and SCRs over time. For the behavioral decisions, there was an interaction between image (sexual vs. neutral) and half (first vs. second) (*F*(1, 33) = 5.78, *p* = .022, partial *η*^2^ = .15). There were significantly more gambles in the sexual (M = .50) than in the neutral (M = .45) image trials for the second half (*F*(1, 33) = 7.56, *p* = .010, partial *η*^2^ = .19) but not for the first half of trials (*p* = .37). The physiological data showed that there was no difference in SCR to outcome of gambling between the first and second half of trials (*p =* .65), indicating no habituation of physiological arousal responses over time. There was no interaction between the factors of image and half (*p =* .65). There was however a main effect of image: the SCRs to gambling outcomes were significantly lower in sexual image trials than in neutral image trials in both the first and second half of the trials (*F*(1, 29) = 4.90, *p* = .035, partial *η*^2^ = .15).

Finally, the SCRs time-locked to the onsets of sexual and neutral images within the time window of 0.5–5 s were analyzed. No significant difference was found between sexual and neutral image trials.

## Discussion

Consistent with prior studies [10, 13, 15], participants in our study showed loss aversion behaviorally: our participants had low proportions of gambles at the trials with risk ratios of .75 and higher, indicating an overweighting of losses relative to gains. The slowest reaction in trials with a risk ratio of .75 indicated larger mental conflicts experienced by participants in deciding whether to gamble or not [25], compared to trials with other risk ratios. Physiologically, participants’ SCRs in response to gambling losses were significantly higher than those to gambling gains. The increased arousal to losses relative to gain is consistent with previous findings (e.g. [17]) suggesting that people have increased cognitive investment with losses compared to gains, resulting in heightened attention and arousal responses. Importantly, we found that sexual stimuli reduced the arousal difference between losses and gains. In our experimental design, the time gaps between the sexual/neutral images and the gambling outcomes were set to be long (about 10–12 seconds) such that the SCRs triggered by the images had returned to baseline before the gambling outcomes were released to the participants. This was confirmed by the statistical result showing that the skin conductance levels at the onset of the gambling outcome were similar in sexual and neutral image trials. Therefore, the smaller differences in SCRs to gambling losses and gains in sexual than in neutral image trials were not due to any baseline differences because of the images presented. An alternative possibility is that the positive emotions triggered by sexual stimuli, which were associated with release of endogenous opioids [26], led to a euphoric state among the participants, which may in turn buffer against the negative emotional arousal triggered by gambling losses. Another possibility is that sexual images compete with gambling losses for attention and arousal responses. The presence of a sexual image might have caused the losses to be less salient for attention. Also, a previous excitation of the sympathetic nervous system by a sexual image (a positive stimulus) may cause a higher threshold for subsequent excitation by a gambling loss (a negative stimulus), causing people to become aroused less easily. The additional source of attention and arousal competition between sexual image and gambling outcome was absent in neutral trials. Further studies are needed to examine this possibility.

Another important observation demonstrating the impact of sexual stimulus on decision-making was that participants’ physiological responses to losses relative to gains predicted gambling decisions only for neutral image but not for sexual image trials. The pattern of results found in neutral image trials suggested that physiological arousal guides decision-making behavior [30, 31], which is congruent with the classic somatic marker hypothesis [32]. Interestingly, in the sexual image trials, physiological responses to monetary losses relative to gains did not predict behaviors, suggesting a decoupling between physiological states and behaviors. The results could be explained by the attentional model of loss [18]: sexual stimuli may oppose the attention enhancement effect of gambling losses, leading to distraction from the task requirements [19, 20] and deviation from the decision algorithm based on outcomes.

In terms of behavioral findings, however, our data did not support the first hypothesis that participants would have a higher proportion of gambling decisions in the sexual than in the neutral image trials for all risk ratios. The effect of sexual stimuli on gambling decisions was close to significance (after corrections for multiple comparisons) only for the riskiest trials (when the risk ratio = 1.5). Our hypothesis was based on the evidence that sexual stimulation facilitates dopamine transmission and enhances activity in the nucleus accumbens (NAcc) [8, 33], which is thought to reflect reward “wanting”. Additionally, greater NAcc activity is related to a stronger saliency of incentives [34] such as money. Our results may imply that the activation of the reward system by sexual stimuli was not sufficient to counteract the effect of loss aversion in low-risk gambling trials. However, in high-risk gambling (such as when risk-ratio = 1.5), participants’ decision may be more susceptible to the effect of sexual stimuli, because of the level of testosterone, which is found to increase under conditions of sexually arousal [3]. Testosterone level is positively associated with risk-taking behaviors in financial decision-making [35–38]. Our finding at trials with a risk-ratio of 1.5 was only close to significance and further study may require a larger sample and trials with a wider range of risk ratios (up to 2) to examine the effect of sexual stimuli in high-risk gambling.

Finally, the SCRs time-locked to onsets of sexual and neutral images were not significantly different. We believe that this was because the financial decision task was presented soon after the images, which was within the time window (0.5–5 s) in which the SCRs to images were measured (Fig 2). The arousal triggered by the financial decision task overlapped with the arousal triggered by the sexual images, thereby reducing the differences between trials with sexual and neutral images. The financial decision tasks were temporally close to the images to ensure that the impact of sexual images was strong during decision-making. However, this might have excluded the possibility of finding the SCR difference between sexual and neutral image trials due to the subsequent financial decision task within the time-window of SCR measurement. Although it is unknown whether the sexual images had indeed triggered an increased SCR compared to neutral images, the participants’ behavioral responses in the picture categorization task indicated that sexual emotions were triggered by the sexual images. Moreover, the finding that there was a significant effect of the sexual image on participants’ physiological responses to gambling outcome also provided evidence that the sexual arousal manipulation was effective. Further study may include a 7-point Likert scale for sexual stimulus rating and a separate session for measuring SCRs to sexual images (without the financial decision task) for checking the effectiveness of sexual arousal manipulation.

### Conclusions and implications

This study demonstrates the interplay between emotion and cognition by showing that viewing sexual images reduces physiological arousal responses to monetary loss and eliminates the association between physiological arousal response to losses relative to gain and gambling decisions. The literature on emotional processes in decision-making reveals that in some circumstances, integrating emotional information into the decision process facilitates optimal decision-making under uncertainty, such as the ability to select advantageous choices in the Iowa Gambling Task [32]. In other scenarios, emotions can dampen logical consistency in decision-making [39], leading to irrational decisions that may bear negative consequences, such as engaging in life-threatening activities [3]. Knowledge about the specific roles of sexual stimulation on financial decision-making would enable people to take an active effort to reduce the negative impact of emotions, for example, by cognitive appraisal [12]. Our findings will be of practical significance to apply in daily life settings that involve financial decision-making. For example, people should be aware that sexual arousal could reduce their attention and physiological sensitivity to monetary losses. In other words, people should pay extra attention to the losses and gains of financial decisions when they are sexually aroused. Some common daily life scenarios include gambling in a casino in the presence of a sexually appealing partner, or gambling through the Internet or purchasing stocks after watching erotic advertisements.

### Limitations

Our study only examined the effect of sexual images and we cannot rule-out the possibility that emotional arousal triggered by other types of activities, such as extreme sports, could influence physiological arousal and decision-making similarly to sexually arousing images. Further studies would be necessary to examine this possibility.

In the post-experiment interview, twenty-four out of the 34 participants reported that they believed the purpose of this experiment was to examine the impact of sexual pictures on their financial decision-making. It is possible that the participants had actively applied cognitive strategies to regulate their own emotions in order to reduce the impact of sexual arousal on their decision-making. Therefore, the behavioral and physiological effects found in our study might be attenuated because of participants’ cognitive regulation. An alternative possibility is that participants were aware of the research objective and acted accordingly to our hypotheses. We believe that this is very unlikely because this act would affect their monetary gains from the gambling task, and monetary incentive was the reason that they participated in this study. Therefore, we believe that the impact of the participants’ conscious awareness of the research objective was a reduction in the effects found. Further, our finding of lowered physiological responses to gambling loss in the sexual image trials reflected the effect of sexual stimulation regardless of participants’ conscious awareness of the research objective. It is still unknown, however, to what further extent sexual stimuli may influence financial decision-making when participants are not consciously aware of the research objective. Further study may attempt to apply different experimental designs, ideally including a naturalistic setting, to examine the research questions while participants’ awareness is minimized.

## Supporting information

S1 File (DOCX)

S1 Dataset (XLSX)

S2 Dataset (XLSX)
