# Supplementary material for: Viewing sexual images is associated with reduced physiological arousal response to gambling loss
Source: PLoS One. 2018 Apr 12;13(4):e0195748. doi: 10.1371/journal.pone.0195748 (PMC5896982; doi:10.1371/journal.pone.0195748)
Supplement: S1 File — (DOCX) [file pone.0195748.s001.docx]

**Instructions for Participants**

In this experiment, you are required to make decisions in two tasks. In the picture categorization task, you will see pictures of women. If you think the picture is sexy, please press the “+” button (on the response box). If you think the picture is neutral, please press the “0” button. Please respond within 1.5 seconds.


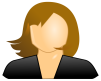


**+ Sexy**

**0 Neutral**

The second task is a financial decision making task. You will first receive $50 cash from the experimenter. Please put it in your wallet. After the experiment is completed, we will randomly select 8 trials out of the total of 88 trials to calculate the amount you win or lose. If the calculated amount is positive, the total amount of money you receive from this experiment will be $50 (the cash you received at the beginning) plus the additional money you win from the financial decision making task. If the calculated amount is negative, the total amount of money you receive will be $50 (the cash you received at the beginning) minus the amount you lose from the financial decision making task. If the amount you lose is more than 50 dollars, you will get zero. According to this calculation scheme, the maximum possible amount you can get is $586 and the minimum possible amount is $0.

In each trial, you have two choices. The figure below is an example.

If you choose the left option, there is a 50% chance that you will obtain the amount of money shown at the upper left corner (i.e. $100). However, there is also a 50% chance that you will lose the amount shown at the lower left corner (i.e. $25).

If you choose the right option, you won’t gain or lose any money (i.e. $0). Please make your decision and respond within 4.5 seconds.


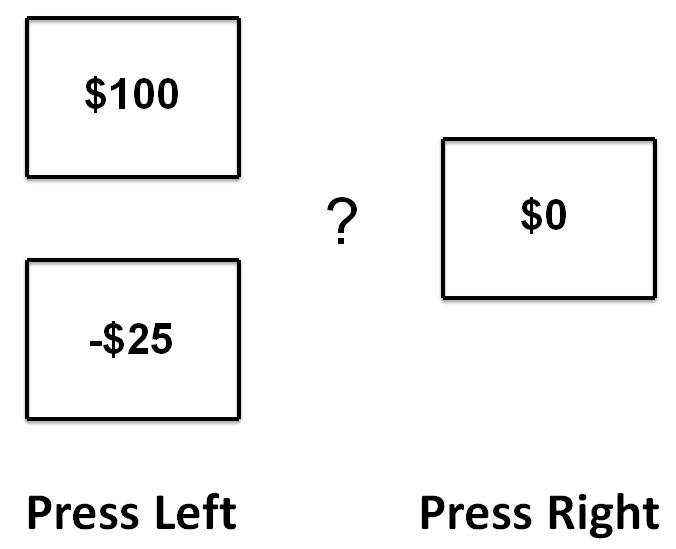


Below is a flow diagram of the financial decision making task. In the example below, the participant chose the left option. In this trial, the outcome is a gain of $100.


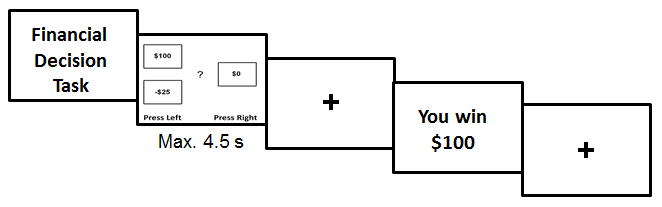


In a small portion of trials, if you choose the left option, you will have a 50% chance to gain the amount shown at the upper left corner. And there is a 50% chance to gain zero (shown at the lower left corner). If you choose the right option, you will certainly (have a 100% chance) gain the amount shown at the right side.


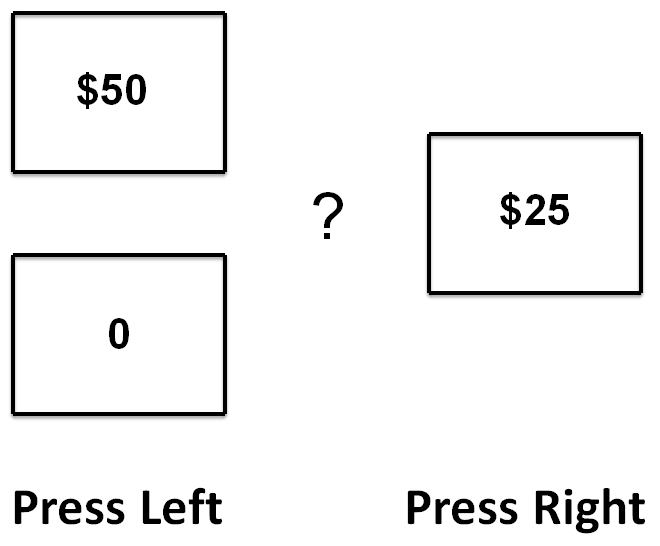


If you understand the experimental instructions, please tell the experimenter what you should do.
